# The FRAILMar Study Protocol: Frailty in Patients With Advanced Chronic Kidney Disease Awaiting Kidney Transplantation. A Randomized Clinical Trial of Multimodal Prehabilitation

**DOI:** 10.3389/fmed.2021.675049

**Published:** 2021-05-19

**Authors:** María José Pérez-Sáez, Andrea Morgado-Pérez, Anna Faura, Elena Muñoz-Redondo, Miguel Gárriz, Maria Dolors Muns, Xavier Nogués, Ester Marco, Julio Pascual

**Affiliations:** ^1^Department of Nephrology, Hospital del Mar, Parc de Salut Mar, Barcelona, Spain; ^2^Department of Physical Medicine and Rehabilitation, Parc de Salut Mar (Hospital del Mar-Hospital de l'Esperança), Barcelona, Spain; ^3^Rehabilitation Research Group, Hospital del Mar Research Institute, Barcelona, Spain; ^4^Institute of Neuropsychiatry and Addictions, Parc de Salut Mar, Barcelona, Spain; ^5^Department of Endocrinology and Nutrition, Hospital del Mar, Parc de Salut Mar, Barcelona, Spain; ^6^Department of Internal Medicine, Hospital del Mar, Parc de Salut Mar, Barcelona, Spain

**Keywords:** chronic kidney disease, exercise, frailty, kidney transplantation, nutrition, prehabilitaion

## Abstract

**Introduction:** Frailty is very frequent among patients with chronic kidney disease (CKD) who are awaiting deceased donor kidney transplantation (KT), and transplant outcomes are worsened in those frail recipients. Frailty and poor fitness powerfully predict mortality, kidney graft survival, and healthcare utilization after KT. Intervention is essential to improve survival and quality of life for frail CKD patients, regardless of their age. Studies of post-transplant physical therapy intervention have been met with limited success, in large part due to high dropout rates. A pre-transplant clinical framework for multimodal prehabilitation interventions including physical therapy, nutritional measures, and psychological support scheduled during the KT waiting list period may improve patient retention and compliance, better mitigate the effects of frailty and poor fitness after KT, and improve main outcomes in frail CKD patients.

**Main Objective:** To study the effectiveness, feasibility, and safety of multimodal prehabilitation (exercise, nutritional plans, psychological advice) in KT candidates.

**Methods:** Randomized controlled clinical trial in 38 frail and 76 non-frail KT candidates. The prehabilitation program will consist of physical exercise (24 sessions, 8 weeks), nutritional supplementation, and psychological advice. The primary endpoint will be a composite achievement of clinical and functional main outcomes in frail and non-frail KT candidates at 90 days post-transplantation. Secondary outcomes include changes in exercise capacity, physical activity, gait speed, respiratory and peripheral muscle strength, muscle size, body composition, performance in activities of daily living (basic and instrumental), anxiety and depression symptoms, and health-related quality of life. Feasibility of the intervention will be also analyzed.

**Expected Results:** Multimodal prehabilitation is a feasible and effective intervention to decrease bad outcomes at 90 days post-KT by 25 and 12.5% in frail and non-frail patients, respectively.

**Clinical Trial Registration:** clinicaltrials.gov (NCT04701398), date: 2021, January 8th (Protocol version: Frailmar_vers2).

## Introduction

### Background and rationale

Frailty is common among patients with chronic kidney disease (CKD), with a prevalence ranging from 7 to 42% among the non-dialysis population (1, 2) and reportedly around 73% in patients on haemodialysis (HD) (3, 4). Patients who are frail experience a decline in physical function and are at increased risk of adverse health outcomes, disability, and mortality (4–8). Candidates to kidney transplantation (KT) have a substantial burden of frailty, with a prevalence of around 20% (9, 10). Furthermore, frailty in KT candidates is associated with poor outcomes post-transplant (9, 11–14).

Efforts to improve outcomes in KT recipients have typically focused on post-transplant interventions. As candidates often have to wait months to years for a deceased donor or spend months identifying an appropriate live donor, pre-transplant intervention may be a uniquely fruitful avenue to explore (1). Moreover, pre- and post-KT physical activity is low among patients with end-stage renal disease (ESRD) (2). This inactivity is associated with poor graft and patient outcomes, including reduced cardio-respiratory fitness, impaired metabolic and nutritional status, reduced quality of life, and increased mortality (15, 16). While awaiting KT, candidates experience a profound loss of functional capacity due to the combination of aging, chronic conditions, and higher risk of frailty in addition to the stress of undergoing HD (17). By the time of KT, there is a high burden of patients with compromised physiology who cannot withstand the stressor of a major surgical intervention. Intervening through exercise training post-KT might not be optimal, given the steep decline in physical activity in the 1st year post-KT and poor compliance with prescribed rehabilitation therapy, as demonstrated through the high dropout rates in two published trials of post-KT exercise (18, 19).

In contrast, KT candidates might be more motivated to exercise knowing that they will be undergoing a major surgery in the coming months; in fact, patients and their providers strongly support prehabilitation in ESRD patients, particularly for vulnerable candidates such as those who are frail (20). Previous randomized controlled trials testing exercise interventions in patients with ESRD and KT recipients have been shown to improve graft function, quality of life, self-reported physical function, aerobic fitness, exercise capacity, and muscle strength, as well as reduced body fat and cardiovascular risk (16, 21–23).

Prehabilitation encompasses exercise-based therapies aimed to enhance preoperative functional capacity and improve tolerance for the upcoming stressor, resulting in better outcomes after the surgery (24). Prehabilitation increases physical activity and improves functional capacity before a major surgical stressor, contributing to a reduction of postoperative recovery time and a quicker return to functional ability (25–27). The prehabilitation interventions include exercise, nutrition, and anxiety-reduction elements. Programs to enhance recovery after surgery are aimed to improve patient outcomes and speed up recovery without compromising safety. In the only study published in KT recipients, enhanced recovery benefited both types of KT procedures (living and deceased grafts), improving patient satisfaction and reducing the length of the hospital stay (28).

Only one small study has assessed the possibility of prehabilitation for KT candidates (29); the intervention (18 participants) consisted of weekly physical therapy sessions at an outpatient center with at-home exercises. With 2 months of prehabilitation, participants improved their physical activity by 64% (*p* = 0.004) based on accelerometry data. Among five prehabilitation participants who received KT during the study, length of stay was shorter than for age-, sex-, and race-matched controls. There was a high level of satisfaction with the prehabilitation intervention, and participants overall reported that it was helping them prepare for KT. Participants noted an increase in physical function and energy, a sustained endurance, a means of weight control, and an improved attitude (29). Given the clinical relevance, it is mandatory to confirm these findings in a larger study with more KT recipients receiving prehabilitation.

Frailty is very frequent among CKD patients included in the waitlist for deceased donor KT, and post-transplant outcomes are worsened in those frail recipients. Moreover, frailty and poor fitness powerfully predict mortality, kidney graft survival, and healthcare utilization after KT. Thus, we hypothesize that a pre-transplant clinical framework for multimodal prehabilitation interventions including physical therapy, nutritional measures, and psychological support could improve patient retention and compliance, better mitigate the effects of frailty and poor fitness after KT, and improve main outcomes in frail CKD patients.

The main objective of this study is to assess effectiveness, feasibility, and safety of multimodal prehabilitation (exercise, nutritional plans, and psychological advice) in KT candidates in the context of a randomized controlled clinical trial. There are three specific objectives: (1) to study the potential effects of multimodal prehabilitation as a prognostic variable to predict the 90-day primary endpoint based on clinical and functional outcomes achieved in frail and non-frail KT candidates; (2) to assess the feasibility and safety of a multimodal prehabilitation program, with especial emphasis on the exercise component in KT candidates; and (3) to assess differences in exercise capacity, muscle function, functional status, and health-related quality of life in frail and non-frail candidates at 1-year post-KT follow-up.

## Methods and Analysis

### Trial Design

The FRAILMar Study is a controlled randomized clinical trial in candidates to KT assigned to the intervention group (multimodal prehabilitation) or control group (standard medical care), with an allocation ratio of 1:1, in a superiority framework. Results are stratified by frail and non-frail patient status. The study protocol is described following the Standard Protocol Items: Recommendations for Interventional Trials (SPIRIT) Statement (3).

### Study Settings

The Departments of Nephrology, Physical Medicine and Rehabilitation, Internal Medicine, Endocrinology, and Psychiatry of the Parc de Salut Mar Consortium (Hospital del Mar and Center Fòrum-Hospital del Mar), Barcelona, Spain will be involved in the trial. Members of the FRAILMAR Study Group are listed in the Appendix, by setting.

### Eligibility Criteria

Adult patients included in the waitlist for deceased donor KT from January 2020 to December 2022 will be eligible. Patients with known chronic muscle diseases and/or unable to perform the exercise plan will be excluded.

### Interventional Methods

The Multimodal Prehabilitation will be focused on behavioral change and the processes that trigger activity, combining physical exercise and motivation toward activity sessions to encourage the practice of moderate regular exercise and healthy habits. All patients in the intervention group, regardless of frailty status, will carry out the prehabilitation program of physical exercise, nutritional supplementation, and psychological advice.

#### Physical Exercise

The exercise component will consist of 1-hour sessions, three times per week for 8 weeks (total 24 sessions). Two of the weekly sessions will be supervised by a physical therapist and the third will be performed unsupervised at the patient's home. Patients will choose between on-site or home-based exercise with the physical therapist; in the case of exercising at home, patients will receive a training kit (cycle ergometer, weights, elastic bands, respiratory muscle trainer); the therapist will telematically supervise the sessions using an internet video call platform. Time schedules for conduction the intervention are detailed in Table 1.

**Table 1:** Time schedules for conducting the intervention.

	**Mon**	**Tues**	**Wed**	**Thurs**	**Fri**	**Sat**
Mornings	Supervised		Supervised		Unsupervised	
Afternoons	Supervised		Supervised		Unsupervised	
Mornings		Supervised		Supervised		Unsupervised
Afternoons		Supervised		Supervised		Unsupervised

All patients will be given access to audiovisual material to support the unsupervised sessions. Each training session will consist of four elements: (1) warm-up (5 min); (2) continuous aerobic training performed on a cycle ergometer (20 min at 60% workload peak, increasing by 5 watts weekly if tolerated); (3) muscle strength exercises of upper and lower limbs (three sets of 10 repetitions of biceps curls, back and shoulder presses, hip flexion and knee extension, and self-weight squats; initial workload will be of 0.5 Kg and increase by 0.5 Kg weekly if tolerated); (4) inspiratory muscle training [five sets of 10 repetitions followed by 1–2 min of unloaded recovery breathing, once a day, 7 days a week for 8 weeks, using an inspiratory muscle trainer (Orygen Inspiratory One-way Valve, Forumed, Spain) at a rate of 15–20 breaths/min; training loads will be set at 30% of maximal inspiratory pressures (PImax) and adjusted weekly by 10 cmH_2_O if tolerated]; and (5) muscle stretching cool-down (5 min).

After completing the 8-week sessions, patients will be encouraged to continue exercising at home or community sports facilities, and will receive coaching from the physical therapist once a month till KT. All study participants shall be removed from the KT waitlist during this period, and re-included after the 12-week assessment, respecting their original placement on the waitlist.

#### Nutritional Supplementation

Well-nourished patients with normal weight or overweight will receive advice to stabilize diet or improve habits, respectively, and just after every exercise session lyophilized protein supplement (20 g) will be administered; once the 8-week supervised intervention is completed, patients will be given a recommendation to drink home-made protein shakes after non-supervised exercise. Diet supplements (protein 20 g + 300 kcal) will be prescribed to patients with severe malnutrition (twice daily), moderate malnourished (daily), or overweight (lyophilized protein 20 g once a day) for 8 weeks (induction) and then alternate days (maintenance).

#### Psychological Advice

Patients will attend an on-site or telematic visit of 1 hour led by a clinical psychologist. During this session, patients shall be given the chance to express their fears and expectations about the KT, impact of HD on health-related quality of life, compliance difficulties with medical treatments, and recommended lifestyle behaviors. After completing the physical training component, patients will be invited to participate in the standardized Mindfulness-Based Cognitive Therapy group program (MBCT), led by an expert psychologist. The MBCT program consists of 8 weekly 2-h sessions. Patients will be offered psychological counseling or treatment as needed until KT.

### Standard Medical Care

In addition to the standard medical treatment, patients will be encouraged to follow the World Health Organization global recommendations on physical activity for health in the context of daily, family, and community activities: leisure time physical activity, transportation, occupational (if still working), household chores, games, sports, or planned exercise.

### Criteria for Discontinuing or Modifying Interventions

Serious adverse events (exercise-induced arrhythmias, cardiac arrest, death) as a result of the multimodal intervention are extremely rare, but should this occur, patients will stop the intervention and continue in the “intention to treat” analysis for the primary end-point. Criteria for discontinuing the intervention include concurrent diseases or changes in the therapeutic plan. A reduced exercise tolerance will justify modification of intensity and/or progression of the protocol.

### Strategies to Improve Adherence to the Intervention Protocol

Planned strategies will include comprehensive information on the benefits of exercise, provided by the health professionals in charge of the patient's care; flexibility of the range of times to take the sessions as a way to combine trial participation and HD sessions; a choice between on-site or telematic supervision; and monthly telematic visits conducted by the physical therapist until KT. Once the intervention is completed, patients will be offered the possibility to attend optional reminder training sessions each month. Intervention adherence will be calculated for each participant. Concomitant exercise interventions are not prohibited during the trial, but the patient shall inform to the research team if this occurs.

### Relevant Concomitant Care and Interventions Permitted or Prohibited During the Trial

Any concomitant care, exercise and nutritional interventions are not permitted during the trial.

### Study Outcomes

**Figure 1:**
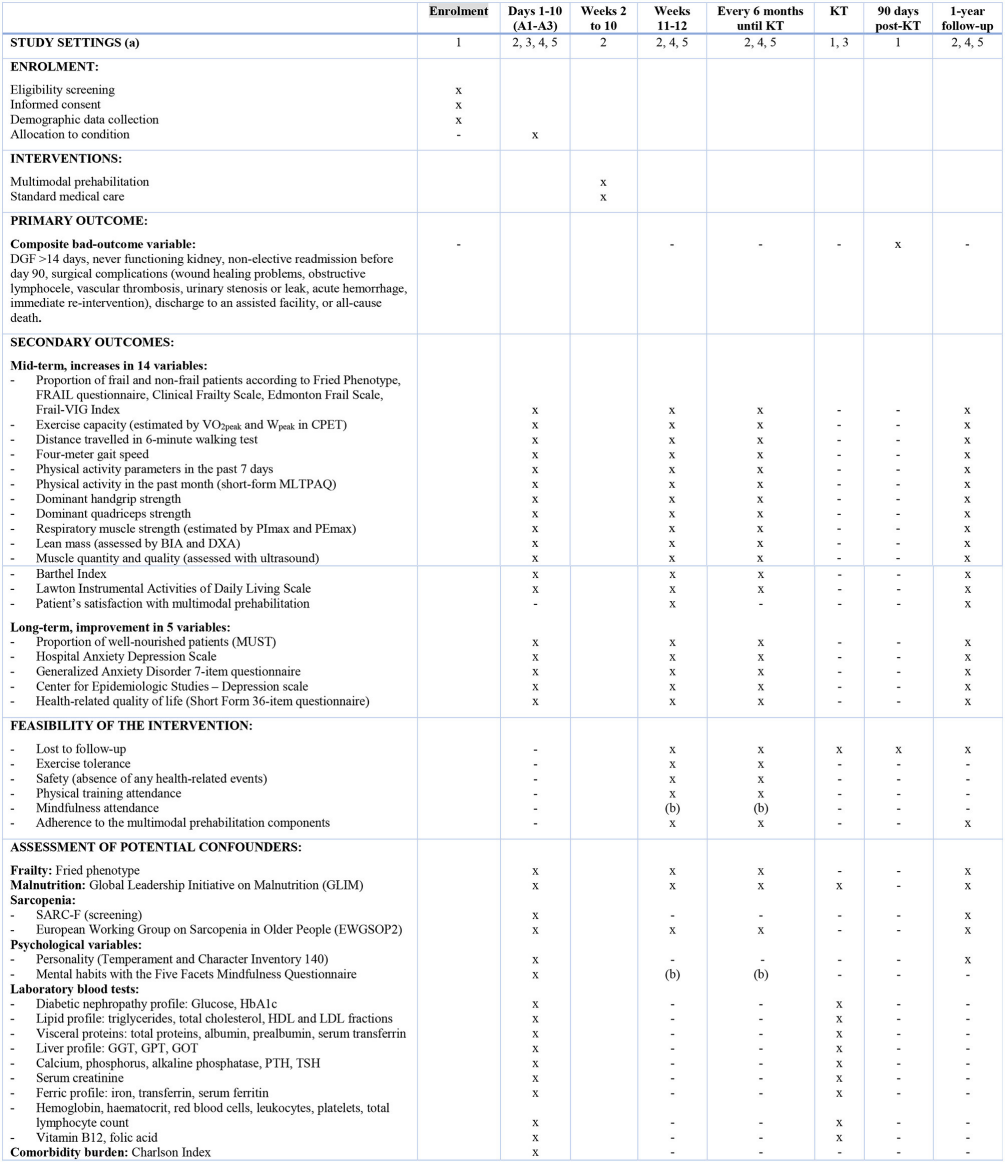
Study settings and schedule of enrolment, interventions, and assessments. (a) Settings: (1) Nephrology Department; (2) Physical Medicine & Rehabilitation Department; (3) Internal Medicine Department; (4) Endocrinology Department; (5) Psychiatric Department. (b) Time of assessment will depend on starting date of the Mindfulness sessions. KT, kidney transplantation; VO2, oxygen uptake; W, workload; CPET, cardiopulmonary effort test; MLTPAQ, Minnesota Leisure-Time Physical Activity Questionnaire; BIA, bioelectrical impedance analysis; DXA, Dual X-ray Absorptiometry; PImax, maximal inspiratory pressure; PEmax, maximal expiratory pressure; SARC-F, Strength, Ambulation, Resistance, Climbing, Falls.

All study outcomes are summarized in Figure 1. The **primary endpoint** will be a bad-outcome composite variable assessed at day 90 post-KT, comprising delayed graft function >14 days, never functioning kidney, non-elective readmission before day 90, surgical complications (wound healing problems, obstructive lymphocele, vascular thrombosis, urinary stenosis or leak, acute hemorrhage, immediate re-intervention), discharge to an assisted facility, or all-cause death.

**Secondary outcomes** will include improvement in mid-term (6 months after intervention) and long-term outcomes (1 year after KT), as well as feasibility and safety variables. The **mid-term outcomes** will include increases in:

- The proportion of non-frail patients at the end of the intervention and during pre-KT follow-up.
- Exercise capacity estimated by peak oxygen uptake (VO2peak) and workload (Wpeak), achieved in a cardiopulmonary effort test (CPET) expressed in mL/Kg/min and watts, respectively. A VO2peak increase of 1.5 mL/Kg/min (minimum clinically important difference) is expected to be achieved after intervention (30).
- Gait speed in a four-meter test.
- Distance traveled in a 6-min walking test (6MWT).
- Physical activity in the past seven days, estimated with accelerometry (total energy expenditure in Kcal, activity-related energy expenditure in MET, number of steps); total activity time will be classified as sedentary, light, moderate, and vigorous according to the Freedson Adult algorithm (31).
- Physical activity in the past month, assessed with a validated questionnaire.
- Peripheral muscle strength estimated with maximal isometric contraction of the dominant hand flexor muscles (in upper limbs) and quadriceps muscles (in lower limbs).
- Respiratory muscle strength estimated with inspiratory and expiratory muscle pressures (PImax and PEmax, respectively).
- Lean mass assessed by dual-energy X-ray absorptiometry (DXA) and bioelectrical impedance analysis (BIA).
- Muscle quantity and muscle quality assessed with the ultrasound parameters recommended by the Sarcopenia through Ultrasound (SARCUS) group in its 2020 update (32).
- Performance in activities of daily living.
- Performance in instrumental activities of daily living.
- Mindfulness dispositional traits.
- Patient satisfaction with multimodal prehabilitation.

Secondary **long-term outcomes** will include improvement in:

- Proportion of well-nourished patients.
- Depression and anxiety symptoms.
- Personality traits.
- Health-related quality of life.

**Feasibility variables** will also be analyzed:

- Losses to follow-up (patients not completing the follow-up assessments).
- Exercise tolerance (patients who adapt to intensity modification).
- Safety, estimated by the absence of any adverse events during the training sessions.
- Physical training participation (yes/no) and attendance (>80% attended sessions or home-based exercise).
- Mindfulness participation (yes/no) and attendance (>80% attended sessions or home-based exercise).
- Adherence to each of the multimodal prehabilitation components (physical activity habits, diet recommendations, and mindfulness habits).

Demographic and clinical characteristics of participants will be recorded. Anticipated variables to be used in the analysis as potential confounders include frailty assessed with the Fried phenotype (33), malnutrition, sarcopenia, comorbidity burden, personality traits, and mindfulness dispositional traits.

All patients will be assessed before and after the supervised exercise intervention, with follow-up every 6 months until KT and 1 year after KT. Time points for each outcome measurement are detailed in Figure 1.

### Participant Timeline

**Figure 2:**
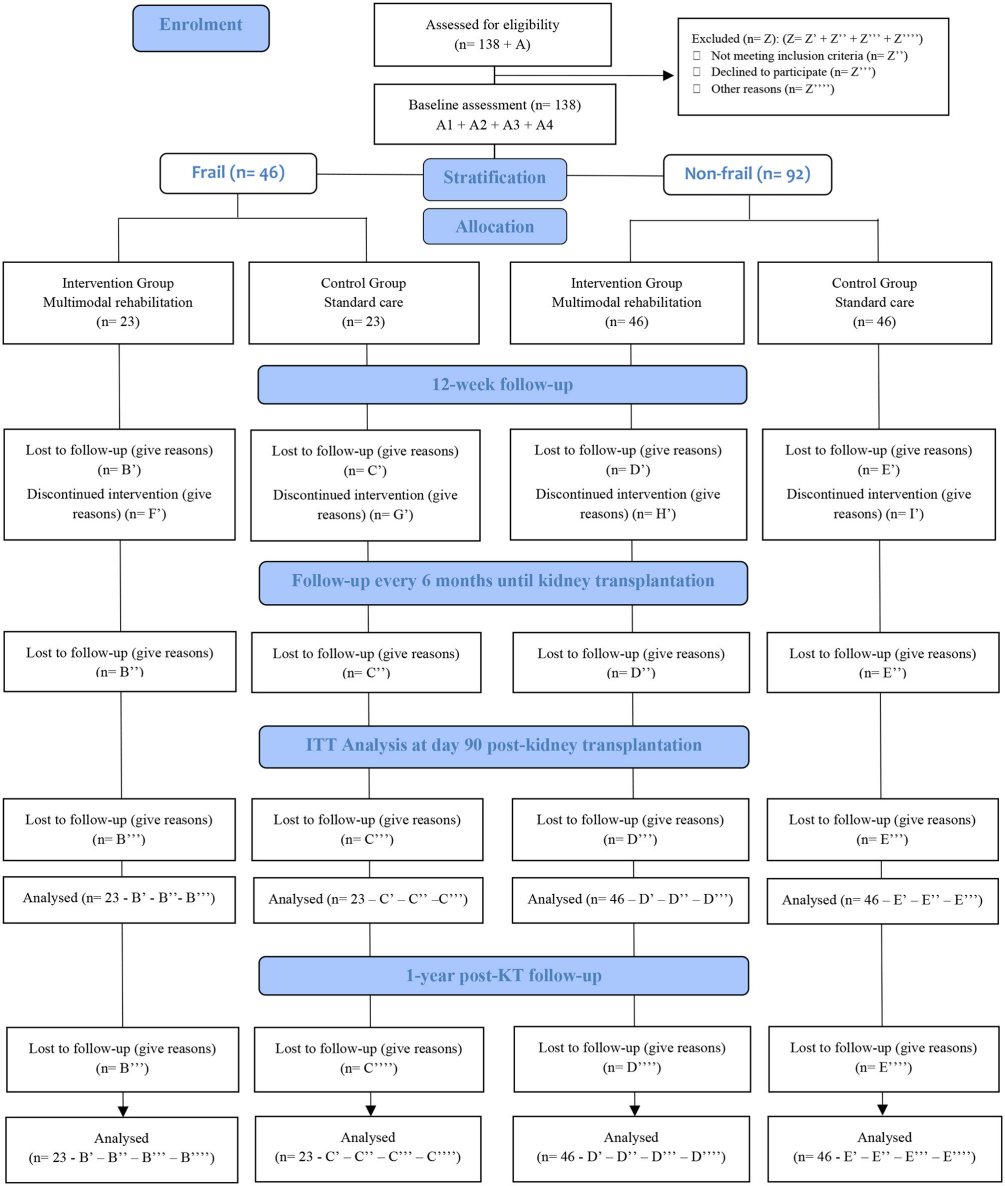
The FRAILMar Study Flow-diagram.

The FRAILMar participant timeline is summarized in Figure 2. Enrolment started in September 2020 and will continue until December 2022. The baseline assessment will be carried out on three different days (A-1, A-2, and A-3). A-1 and A-2 will take place in the Rehabilitation Department and A-3 in the Nephrology, Endocrinology, and Internal Medicine Departments, as shown in Table 2. The study tests and questionnaires will be administered as detailed in Figure 1.

**Table 2:** Baseline assessment: sequence of examinations and delivery of questionnaires.

A-1 Day 1	Clinical visit	• Anamnesis • Charlson Comorbidity Index • Frailty stratification (Fried phenotype) • SARC-F screening tool • Barthel Index • Lawton Instrumental Activities of Daily Living Scale
	Examinations carried out in the Rehabilitation Department	• Bioelectrical impedance analysis (InBody S10) • Muscle ultrasound (forearm, quadriceps, and intercostal muscles) (Esaote MyLabSeven^®^) • Dominant handgrip isometric contraction (Jamar^®^ Plus) • Dominant quadriceps isometric contraction (Mecmesin^®^) • Four-meter gait speed • 6-min walking test • Respiratory muscle pressures (MicroRPM) • Cardiopulmonary effort test
	Delivery of self-administered questionnaires	• Minnesota Leisure-Time Physical Activity Questionnaire (short form) • Malnutrition Universal Screening Tool • Hospital Anxiety and Depression Scale • Generalized Anxiety Disorder seven-item questionnaire • Center of Epidemiological Studies Depression • Five Facets Mindfulness Questionnaire • Temperament and Character Inventory 140 • Short Form 36-item health questionnaire
	Delivery of activity monitors	• Actigraph GT3-x
A-2 Day 7	Frailty measures other than Fried phenotype	• Frail Scale • Clinical Frailty Scale • Edmonton Frail Scale • Frail-VIG Index
	Return of activity monitors	• Actigraph GT3-x
A-3 Day 7–14	Other assessments	• Laboratory blood tests • Dual-energy X-ray absorptiometry (Hologic Horizon Wii) • Nutritional assessment: anamnesis, anthropometry

The first baseline assessment (A-1) will last nearly 3 h and will be divided in three parts:

1. Clinic visit: Frailty assessment, anamnesis and physical examination with clinical history will be carried out by a physiotherapist who will explain the multimodal prehabilitation details and answer patient questions about the trial. The Fried Phenotype will be used to stratify participants as non-frail (0–1 items) or frail (2–5 items) prior to randomization.
2. Battery of tests carried out in the Rehabilitation Department, in sequence: BIA, ultrasound of the intercostal, forearm and quadriceps muscles, isometric dynamometry, four-meter gait speed test, 6MWT, maximal respiratory muscle pressures, and CPET.
3. Delivery of self-administered scales and activity monitors: Immediately after completing the test battery, a set of self-administered questionnaires (malnutrition screening, anxiety and depression symptoms, mindfulness dispositional traits, personality traits, and health-related quality of life) will be delivered to patients to be completed during the following week. Activity monitors to record daily physical activity for the next 7 days also will be delivered.

The second baseline assessment (A-2) will be carried out 1 week later, when patients return the self-administered questionnaires and activity monitors. During this second visit, frailty will be assessed with the use of frailty measurement tools other than the Fried phenotype.

The third baseline assessment (A-3) will consist of laboratory blood tests, DXA, and a visit with a nutritionist. Based on anthropometry, diet history, alimentary habits, nutritional requirements, BIA reports conducted in A-1, and scores from the malnutrition screening tools, patients will be classified as having severe malnutrition, moderate malnutrition, overweight/obesity, and good nutrition status. Further nutritional controls will be scheduled for week 12 and every 6 months until KT.

### Sample Size

Sample size is calculated to identify significant differences in overall bad-outcome in KT recipients. Accepting an α risk of 0.05 and a β of 0.2 in two-sided testing, 38 frail participants (19 in each group, intervention and control) will be necessary to recognize as statistically significant a decrease of 25% in bad outcomes. The sample size has been overestimated to allow potential losses of 20%, requiring a final sample of 46 patients (23 in each group). Similarly, 76 non-frail patients (38 in each group, intervention and control) will be necessary to recognize as statistically significant a decrease of 12.5% in bad outcomes. Given the patient mortality after listing, the potential for exclusion from the kidney transplant waiting list due to medical issues, and the possibility of voluntary drop-out, 20% of potential losses has been considered to estimate a final sample of 92 (46 in each group). Therefore, sample size in the FRAILMar study will be of 138 participants.

### Recruitment

Every year, around 100–120 patients receive a KT from deceased donor in the study setting; thus it is realistic to reach the target sample size during the study period. Strategies for recruiting will be similar to those described to improve adherence to interventions. Efforts will be focused on offering schedule flexibility and involving professionals able to motivate patients and clearly explain anticipated benefits of the multimodal intervention.

### Allocation

**Sequence generation:** A random number generator program will be used to allocate patients to the intervention and control groups for each stratum: non-frail or robust (Fried scores 0–1) and frail (Fried scores 2–5).

**Allocation concealment mechanisms:** Allocation concealment will be ensured as the person in charge of randomization will not release the randomization codes until participants have completed all baseline measurements.

**Implementation:** A clinician blinded to patient identification will generate the allocation sequence, doctors and nurses from the Nephrology department will enroll potential participants, and the physical therapists in charge of assessments A-1 and A-2 will assign participants to the interventions.

### Blinding

As usually happens in exercise-based interventions, participants and therapists cannot be blinded after assignments. Moreover, researchers in charge of interventions will be involved in most assessments; therefore, the FRAILMar study is an open trial except for researchers in charge of data analysis.

### Data Collection, Management and Analysis

#### Data Collection Methods

Data collection sheets will be used by the researchers in charge of collecting the study variables. These researchers will periodically update the database created by one of the principal investigators, and data collection sheets will then be stored according to current regulations. Processes to promote data quality include administration of questionnaires and tests following the same sequence, to minimize intra-rater reliability by using the mean value of three reproducible measures (e.g., muscle ultrasound parameters), and use of the highest value of three reproducible measures for outcomes requiring volitional maneuvers. Moreover, the researchers in charge of assessments will carry out a 3-day training on standardized protocols. To promote retention of participants, the physical therapists will coach participants through the follow-up period and will make telephone contact in the days before each assessment.

#### Study Instruments

**Frailty measures:** (a) Fried Phenotype: The five frailty criteria described by Fried are weight loss, exhaustion, low physical activity, slowness, and weakness; their sum score classifies individuals as not frail (score 0), pre-frail (score 1–2) and frail (score 3–5) (32); however, in renal populations patients have been categorized as follows: 0–1 as robust, two pre-frail and ≥3 frail. In addition, pre-frail and frail categories are usually joined and those patients are considered as frail; (b) FRAIL Scale: Five yes-no items addressing fatigue, resistance (cannot climb one flight of stairs), aerobic (cannot walk one block), illnesses (more than 5), and loss of weight (more than 5% in the last 6 months); patients are classified in robust (score 0), pre-frail (score 1–2), and frail (score 3–5) (34); (c) Clinical Frailty Scale: This is a nine-point scale that classifies individuals from very fit (score 1) to terminally ill (score 9) (35, 36); (d) Edmonton Frail Scale: This 12-item scale covers nine frailty domains (cognition, general health status, functional independence, social support, medication use, nutrition, mood, continence, and functional performance); the total score ranges from 0 to 17 and classifies patients as non-frail (score 0–5), vulnerable (score 6–7), mild frailty (score 8–9), moderate frailty (score 10–11), and severe frailty (score 12–17) (37); and (e) Frail-VIG index: This is a scale ranging from 0 to 1, where scores from 0.2 to 0.25 indicate frailty (38).

**Cardiopulmonary effort test:** The incremental CPET will be conducted on a cycle ergometer connected to a gas analyser and a 12-lead electrocardiogram, using blood pressure and pulse oximeter monitoring. Measurements will be taken at rest, pedaling without any resistance for 2 min, pedaling against a continuously increasing resistance, and in the recovery phase 2 min after exercise according to recommendations by the American Thoracic Society (ATS) (39). A CPET computer software package will be used; predicted normal values will be calculated.

**Six-min walking test:** The test will be performed along a 30-m flat straight corridor for 6 min according to ATS testing standards (40), using the VyntusTM Walk (Vyaire Medical, California, US) system.

**Accelerometry:** Participants will be requested to wear an adjustable cotton fabric wristband containing the Actigraph GT3-x monitors for 7 consecutive days (31, 41).

**Minnesota Leisure-Time Physical Activity Questionnaire (MLTPAQ):** This is a valid and reliable questionnaire to quantify energy expenditure during leisure time in persons older than 50 years. The MLTPAQ consists of six questions administered in an interview of around 5 min and has been validated for the Spanish population (42).

**Four-meter gait speed test:** Gait speed will be tested according to standardized methods at usual pace in a four-meter corridor; gait speed <0.8 m/s is considered reduced (43).

**Isometric dynamometers:** The Jamar^®^ plus hand dynamometer (Performance Health Supply, Cedarburg, USA) and the digital dynamometer Mecmesin AFG 500N (Mecmesin, West Sussex, United Kingdom) will be used to determine maximal isometric contraction of finger flexor and quadriceps muscles, respectively. The highest value of three reproducible maneuvers (<10% variability between values) will be used for analysis following standardized methods (44, 45).

**Respiratory muscle pressure meters**: PImax will be measured at mouth during a maximum effort from residual volume to an occluded airway; PEmax will be determined from total lung capacity in the face of the occluded airway by using the MicroRPM pressure transducer (MicroMedical/Carefusion, Kent, United Kingdom) and expressed as a percentage of the reference values determined for a Mediterranean population (46).

**Bioelectrical impedance:** BIA parameters will be assessed with the InBody S10 device (InBody Co. Ltd, Seoul, Korea).

**Dual X-ray Absorptiometry:** Total body DXA will be determined using the Hologic Horizon Wi, (Hologic^®^, Marlborough, USA) device for the measurement of body composition (fat mass and muscle mass).

**Muscle ultrasound (dominant forearm, quadriceps, and intercostal muscles): Muscle** quantity and quality according to the SARCUS recommendations (47) will be assessed using B-mode on the Esaote MyLabSeven imaging system (Esaote, Genoa, Italy) with a 5-cm wide 7.5-MHz linear probe.

**Barthel Index:** Ordinal scale that measures function independence in feeding, moving from chair to bed, grooming, transferring to and from a toilet, bathing, walking on a level surface, going up and down stairs, dressing, and continence. The score of each of the items are summed to create a total score ranging from 0 to 100, with lower scores indicating more dependence (48).

**Lawton Instrumental Activities of Daily Living Scale:** Instrument developed to assess independent living skills; there are eight domains of function and scale range goes from 0 (low function, dependent) to 8 (high function, independent) (49).

**Satisfaction scale:** A five-level Likert scale created by researchers for this study. Patients shall indicate a response to three statements on the basis of a 5-level Likert scale: (1) I am very satisfied with the overall prehabilitation program; (2) I think this program has helped me to improve my physical status; and (3) It has been easy to combine the prehabilitation program with my daily life.

**Malnutrition Universal Screening Tool (MUST):** This is a five-step screening tool to identify malnutrition, risk of malnutrition, and obesity based on body mass index, weight loss, and harmful effect of acute disease on meeting oral nutrition requirements for more than 5 days (50).

**Hospital Anxiety Depression Scale:** The HADS consists of two seven-item subscales assessing two domains (anxiety and depression, respectively). The Spanish version of the HADS has demonstrated reliability and validity as a screening tool for psychiatric disorders in general hospital outpatients. A cut-off point of five showed adequate sensitivity (77.8%) and specificity (80.9%) for the depression subscale, and a cut-off point of eight showed adequate values of sensitivity (88.9%) and specificity (77.2%) for the anxiety scale (51).

**Generalized Anxiety Disorder-7 (GAD-7):** A simple seven-item tool to detect cases of generalized anxiety disorders. The adapted version for the Spanish population has a one-dimensional structure that matches the original structure based on the DSM-IV diagnostic criteria. A cut-off value of 10 showed adequate values of sensitivity (86.8%) and specificity (93.4%) (52).

**Center of Epidemiologic Studies-Depression (CES-D):** A self-rating scale composed of 20 items, designed to detect depressive symptomatology. It has been extensively used in ESRD (53). A cut-off value of 16 has shown adequate sensitivity (0.95) and specificity (0.91) in the Spanish validation study (54).

**Short Form 36 health questionnaire:** The SF-36 consists of eight domains yielding two summary measures: physical and mental health. The physical component includes four dimensions (physical functioning, role-physical, bodily pain, and general health); the mental health score is composed of vitality, social functioning, role-emotional, and mental health (55); the SF-36 has been validated for use in the general Spanish population (56).

**GLIM criteria:** The GLIM consensus is a three-step approach to assess malnutrition where patients are first identified by any validated screening tool (e.g., MUST) and then diagnosed if they meet at least one phenotypical characteristic (non-volitional weight loss, low body mass index, or reduced muscle mass) and one etiological criterion (reduced food intake or assimilation, disease burden, or inflammation); finally, severity is determined, based on threshold levels of the phenotypic criteria (57).

**SARC-F:** The SARC-F is a questionnaire to detect individuals who may have sarcopenia based on the assessment of five domains: strength, walking across a room, rising from a chair, climbing stairs, and number of falls in the last year. Each item is scored at one of three subjective levels of difficulty. The questionnaire has been validated for the Spanish population (58).

**European Working Group on Older People 2020 updated criteria (EWGSOP2):** The updated EWGSOP definition of sarcopenia is based on the presence of low muscle strength and low muscle mass (59).

**Temperament and Character Inventory-140 (TCI-140):** The TCI-140 consists of 140 items, of which 136 relate to the seven temperament and character domains (novelty seeking, harm avoidance, reward dependence, persistence, self-directiveness, cooperativeness, and self-transcendence), and the remaining four items measure response accuracy/carelessness. The TCI-140 represents a short form of the original Temperament and Character Inventory-Revised (TCI-R; 240 items) and has shown adequate reliability (alpha from 0.72 to 0.82) except for novelty seeking (alpha = 0.63) (60).

**Five Facets Mindfulness Questionnaire (FFMQ):** This 39-item self-administered questionnaire measures five facets of mindfulness (Observing, Describing, Acting with awareness, Non-judgmental, and Non-reactive); the Spanish version has shown adequate internal consistency (alpha ranging from 0.75 to 0.91) (61).

**Charlson Comorbidity Index:** This score was originally developed to evaluate prognosis based on weightings for specific comorbidities; each of these conditions is given a weighting of 1 to 6 and weighted scores are summed (62–64).

#### Data Management

One of the principal investigators (PI) will create the database. Two collaborators will be in charge of entering data under PI supervision and keeping the database uploaded. To ensure participant confidentiality, a registration number will be assigned to each participant. No personal information will be contained in the database. Range checks for data values and regular audits will be conducted to promote data quality.

#### Statistical Methods

Quantitative variables will be presented as mean and standard deviation, unless otherwise stated, and descriptive variables as absolute numbers and percentages. Univariate analysis will be performed using appropriate statistical tests depending on variable distribution. Treatment effect will be analyzed by changes in muscle mass and strength pre- and post-intervention. Changes during follow-up will be assessed by analysis of variance using mixed repeated measures and a one-factor design for the analysis of values over time. The outcomes from the RCT will be analyzed by intention-to-treat and the results over the follow-up period will be reported considering potential scenarios of non-compliance and non-adherence. Additional univariate analyses (e.g., subgroup and adjusted analyses) according to sex, age, and frailty will be performed. Significance level will be set at *p* ≤ 0.05.

#### Data Monitoring

The establishment of a Data Monitoring Committee is not necessary given the study characteristics. No interim analyses will be conducted.

#### Harms

Exercise-related adverse effects (e.g., chest pain, dizziness, skeletal muscle injuries) will be recorded during the training sessions. No other unintended effects related to nutritional supplementation or psychological intervention are expected.

#### Auditing

The principal investigators will be in charge of auditing the trial procedures (assessments and interventions) within the 1st month of the project and every 6 months thereafter until completing data collection.

## Discussion

Frailty in CKD patients displays a unique constellation of features that characterize this special population, including muscle wasting, anorexia, protein energy wasting, inflammation, oxidative stress, catabolic/anabolic hormone imbalance, metabolic acidosis, and other cellular alterations. Intervention is essential to improve quality of life for frail CKD patients, regardless of their age. Studies of post-transplant physical therapy intervention have been met with limited success, in large part due to high dropout rates. A pre-transplant clinical framework that provides multimodal prehabilitation interventions including physical therapy, nutritional measures, and psychological support may improve patient retention and compliance, better mitigate the effects of frailty and poor fitness after KT, and improve main outcomes in frail CKD patients.

Prehabilitation is more challenging in KT than in other surgical settings because the transplantation date is usually unknown. Furthermore, not all KT candidates can combine prehabilitation sessions with their HD schedules. Work schedules and geographic distance can also limit participation. In a recent survey, only 25% of HD patients were able to exercise without difficulty; the major barriers for the remaining patients were feeling too tired (55%), shortness of breath (50%), and too weak (49%) (65). If patients were to exercise, they preferred to exercise at home (73%) using a combination of aerobic and resistance training (41%), regardless of modality or age category. Finally, an extremely large sample size would be required to assess long-term outcomes like death or graft loss, as well as other important clinical outcomes that result from increased physical activity (66–68).

The main limitation is the low physical profile of many frail CKD patients, even those scheduled for KT. Consequently, some patients will not consent to undertake the intervention program and the adherence could be low, with high dropout rates. Efforts will be made by the research team to facilitate all logistical requirements and explain the strong potential benefits to the patients and their families. The process of designing the prehabilitation plan will take account of the final users' perspective in order to achieve a realistic, acceptable, user-friendly approach that covers patient needs and preferences and avoids simplistic solutions that ignore the patients' reality.

## Ethics Statement

The FRAILMar Study protocol and the informed consent have been approved by the local Ethics Committee of the Hospital del Mar Research Institute, Barcelona, Catalonia, Spain (Nr. 019/8623/I). National and international research ethics guidelines, including the Declaration of Helsinki, and current confidentiality law concerning personal data (Spanish Organic Law 3/2018, and European Parliament and Council Regulation EU 2016/619) will be followed. Detailed and understandable oral and written information will be provided, and informed consent to participate will be signed by all participants. Important protocol modifications will be communicated to involved parties (researchers, participants, and trial registry. The principal investigators will have access to the final trial dataset. The trial results will be communicated to participants, healthcare professionals, and patient groups (e.g., scientific meetings, publications in peer-reviewed journals). Informed consent is available in Catalan and Spanish languages upon request.

## Author's Note

This project is part of EM-R's Ph.D. project Effects of prehabilitation in patients awaiting kidney transplantation in the Ph.D. program in Medicine, Medicine Department, Universitat Autònoma de Barcelona, Spain.

## Author Contributions

MP-S, EM, and JP contributed to conception and design of the study. AM-P wrote the first draft of the manuscript. MP-S, EM-R, MG, MM, EM, and JP wrote sections of the manuscript. All authors contributed to manuscript revision, read, and approved the submitted version.

## Conflict of Interest

The authors declare that the research was conducted in the absence of any commercial or financial relationships that could be construed as a potential conflict of interest.

## Abbreviations

ATS: American Thoracic Society
BIA: Bioelectrical impedance analysis
CES-D: Center for Epidemiologic Studies Depression
CKD: Chronic kidney disease
CPET: Cardiopulmonary effort test
DXA: Dual-energy X-ray Absorptiometry
ESRD: End-stage renal disease
EWGSOP: European Working Group on Sarcopenia in Older People
FFMQ: Five Facets Mindfulness Questionnaire
GAD-7: Generalized Anxiety Disorder seven-item questionnaire
GLIM: Global Leadership Initiative on Malnutrition
HADS: Hospital Anxiety and Depression Scale
HD: Hemodialysis
KT: Kidney transplantation
MBCT: Mindfulness Based Cognitive Therapy
MLTPAQ: Minnesota Leisure Time Physical Activity Questionnaire
MUST: Malnutrition Universal Screening Tool
PImax: Maximal inspiratory pressure
PEmax: Maximal expiratory pressure
SARC-F: Strength, Assistance in walking, Rise from chair, Climb stairs, and Falls
SARCUS: Sarcopenia through Ultrasound
SF-36: Short Form 36-item health questionnaire
SNAQ: Subjective Nutritional Assessment Questionnaire
SPIRIT: Standard Protocol Items, Recommendations for Interventional Trials
TCI: Temperament and Character Inventory
VO_2peak_: Peak oxygen uptake
W_peak_: Peak workload
6MWT: 6-min walking test.
