# Supplementary material for: The FRAILMar Study Protocol: Frailty in Patients With Advanced Chronic Kidney Disease Awaiting Kidney Transplantation. A Randomized Clinical Trial of Multimodal Prehabilitation
Source: Front Med (Lausanne). 2021 May 19;8:675049. doi: 10.3389/fmed.2021.675049 (PMC8170320; doi:10.3389/fmed.2021.675049)
Supplement: Supplementary file 1 [file Presentation_1.PDF]

FRAILMar Study Group members, by setting (authors in bold type).

**City**	**Setting**	**Researchers,** ***by department***
Barcelona	Parc de Salut Mar - Hospital del Mar Medical Research Institute	***Department of Nephrology:*** **María José Pérez-Sáez**, Carlos E. Arias-Cabrales, Dolores Redondo, Francesc Barbosa, Higini Cao, Silvia Collado, Maria Dolores Arenas, Anna Buxeda, Carla Burballa, Marta Crespo, **Julio Pascual, Anna Faura,** María Vera, Anna Bach, Guillermo Pedreira, Ernestina Junyent, Montserrat Folgueiras, Yolanda Castillo, Aida Martínez, and Rosa Causadías.<br>***Department of Cardiology:*** Alicia Calvo.<br>***Department of Rehabilitation and Physical Medicine:*** **Ester Marco,** Delky Meza-Valderrama, **Andrea Morgado-Pérez,** and **Elena Muñoz-Redondo**.<br>***Department of Anesthesiology:*** Jesús Carazo.<br>***Department of Urology:*** Albert Frances and Lluis Cecchini.<br>***Department of Internal Medicine:*** **Xavier Nogués**.<br>***Department of Psychology, Neuropsychiatric Institute:*** **Miguel Gárriz** and Maria Polo Gómez.<br>***Department of Endocrinology and Nutrition, Dietary Unit:*** **María Dolors Muns**.<br>***Department of Geriatrics:*** Olga Vázquez.
Barcelona	Fresenius Medical Care	***Diagonal Hemodialysis Center:*** Sara Hurtado.<br>***Glories Hemodialysis Center:*** Laura Ribera.
Granollers	Fresenius Medical Care	***Granollers Hemodialysis Center:*** Josep Mora and Omar Ibrik.
Madrid	Hospital Universitario de Getafe	***Department of Geriatrics:*** Leocadio Rodríguez-Mañas.
Manresa	Hospital de Manresa	***Department of Nephrology:*** Núria Garra and Josep Galcerán.
Mollet	Hospital de Mollet	***Department of Nephrology:*** Ramón Roca and Jordi Calls.
Palamós	Hospital de Palamós	***Department of Nephrology:*** Rosa García.
Sabadell	Hospital Parc Taulí	***Department of Nephrology:*** Thaïs López and Jaume Almirall.
Terrassa	Consorci Sanitari de Terrassa	***Department of Nephrology:*** Fátima Moreno and Manel Ramírez de Arellano.
	Fresenius Medical Care	***Cetirsa Terrassa Hemodialysis Center:*** Ignacio Cidraque.
Vilanova i la Geltrú	Consorci Sanitari del Garraf	***Department of Nephrology:*** Sara Outón and Fabiola Dapena.
